# Mitogenome of *Mytilus trossulus* (Mytilidae, Bivalvia) isolated from a 1920 herbarium specimen

**DOI:** 10.1080/23802359.2016.1181995

**Published:** 2016-07-08

**Authors:** Jeffery R. Hughey, Ga Hun Boo, Sung Min Boo

**Affiliations:** a Division of Mathematics, Science, and Engineering, Hartnell College, Salinas, CA, USA;; b Herbarium, University of California at Berkeley, Berkeley, CA, USA;; c Department of Biology, Chungnam National University, Daejeon, Korea

**Keywords:** Blue mussel, Ceramium, mitogenome, population genetics, shotgun sequencing

## Abstract

DNA was extracted from a red algal herbarium specimen collected in 1920 and subjected to next generation sequencing. Here we report the assembly of the mitogenome of a marine mussel, *Mytilus trossulus*, deciphered from this plant museum specimen. The mitogenome is 16,744 bp in length, contains 38 genes, and is more similar to other *M. trossulus* reported from the Baltic Sea. The data show that in addition to plant DNA, herbarium specimens also contain genetic information from invertebrates that may be valuable for genomic, population and phylogenetic studies of animals.

Whole-genome shotgun sequencing does not discriminate between target and exogenous DNA during the library construction step (Metzker 2010). It is, therefore, not uncommon after the assembly to identify contigs that represent non-targeted species such as bacteria, herbivores or epiphytes. We extracted DNA from a snippet of the lectotype specimen of the marine red algal species *Ceramium cimbricum* H.E. Peterson in Rosenvinge (1924, p. 378) (Ceramiales: Rhodophyta) housed in the Natural History Museum Denmark (C-A-37605) following the protocol of Lindstrom et al. (2011). The library was constructed using the methods described in Hughey et al. (2014) and analyzed on the Illumina HiSeq with 36 bp paired-end sequencing (Illumina Inc., San Diego, CA). The sequencing generated 17,907,810 filtered reads that were assembled with the default denovo settings using CLC Cell 4.3.0 (^®^2015 CLC bio, a QIAGEN Company, Hilden, Germany).

In addition to red algal DNA, analysis of the reads using a Standard Nucleotide Blast search at NCBI identified three overlapping contigs representing the complete mitogenome of *Mytilus trossulus*. The mitogenome of *M. trossulus* (GenBank KU925349) is 16,744 bp in length, AT skewed (61.3%), and contains 38 genes including 23 transfer RNAs (Leucine, Methionine, and Serine are duplicated), seven NADH dehydrogenase subunits (1, 2, 3, 4, 4L, 5 and 6), three cytochrome c oxidase subunits (1, 2 and 3), two ribosomal RNAs (one small and one large), two ATP synthase F0 subunits (6 and 8) and cytochrome b. The mitogenome is classified to haplogroup F, and is highly conserved. It differed in sequence from a female specimen from Puck Bay, Baltic Sea by 14 SNPs and three gaps (GenBank KM192128, Zbawicka et al. 2014), and from another female specimen from the Gulf of Gdańsk, Baltic Sea by 57 SNPs and three gaps (GenBank DQ198231, Burzyński et al. 2006). Alignment of the mitogenome using MAFFT (Katoh & Standley 2013) and analysis with RaxML (Stamatakis 2014) with default settings in Galaxy (Giardine et al. 2005; Blankenberg et al. 2010; Goecks et al. 2010) placed *M. trossulus* in a fully supported clade with nine mitogenomes of *M. trossulus* from the Baltic Sea (Figure 1).

**Figure 1.**
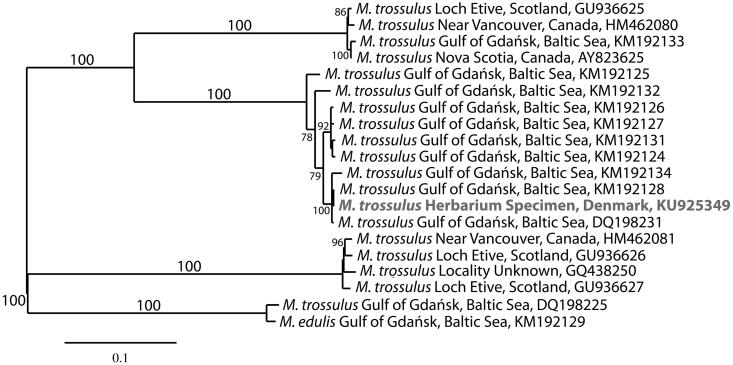
Maximum-likelihood phylogram of representative *Mytilus* mitogenomes. Numbers along branches are RaxML bootstrap support values based on 1000 nreps (<70% support not shown). The legend below represents the scale for nucleotide substitutions.

We are not advocating that zoologists initiate full-scale destructive sampling of valuable herbarium specimens. However, our data indicate that in cases where historic materials of marine invertebrates are needed to address population level, taxonomic, systematic, or the timing of the introduction of an invasive species, plant collections in herbaria may hold crucial genetic information to answer these questions.

## References

[CIT0001] Blankenberg D, Von Kuster G, Coraor N, Ananda G, Lazarus R, Mangan M, Nekrutenko A, Taylor J 2010 Galaxy: a web-based genome analysis tool for experimentalists. Curr Protoc Mol Biol. Ch. 19, Unit 19.10:11–21. 10.1002/0471142727.mb1910s89 PMC4264107 20069535

[CIT0002] Burzyński A, Zbawicka M, Skibinski DO, Wenne R 2006 Doubly uniparental inheritance is associated with high polymorphism for rearranged and recombinant control region haplotypes in Baltic *Mytilus trossulus.* Genetics. 174:1081–1094. 16951056 10.1534/genetics.106.063180 PMC1667088

[CIT0004] Giardine B, Riemer C, Hardison RC, Burhans R, Elnitski L, Shah P, Zhang Y, Blankenberg D, Albert I, Taylor J, et al 2005 Galaxy: a platform for interactive large-scale genome analysis. Genome Res. 15:1451–1455. 16169926 10.1101/gr.4086505 PMC1240089

[CIT0005] Goecks J, Nekrutenko A, Taylor J, The Galaxy Team. 2010 Galaxy: a comprehensive approach for supporting accessible, reproducible, and transparent computational research in the life sciences. Genome Biol. 11:R86. 20738864 10.1186/gb-2010-11-8-r86 PMC2945788

[CIT0006] Hughey JR, Gabrielson PW, Rohmer L, Tortolani J, Silva M, Miller KA, Young JD, Martell C, Ruediger E 2014 Minimally destructive sampling of type specimens of *Pyropia* (Bangiales, Rhodophyta) recovers complete plastid and mitochondrial genomes. Sci Rep. 4:5113. 24894641 10.1038/srep05113 PMC4044621

[CIT0007] Katoh K, Standley DM 2013 MAFFT multiple sequence alignment software version 7: improvements in performance and usability. Mol Biol Evol. 30:772–780. 23329690 10.1093/molbev/mst010 PMC3603318

[CIT0008] Lindstrom SC, Hughey JR, Martone PT 2011 New, resurrected and redefined species of *Mastocarpus* Phyllophoraceae, Rhodophyta from the northeast Pacific. Phycologia. 50:661–683.

[CIT0009] Metzker ML 2010 Sequencing technologies – the next generation. Nat Rev Genet. 11:31–46. 19997069 10.1038/nrg2626

[CIT0010] Rosenvinge LK 1924 The marine algae of Denmark. Contributions to their natural history. Part III. Rhodophyceae III. (Ceramiales). Kongelige Danske Videnskabernes Selskabs Skrifter, 7. Række, Naturvidenskabelig og Mathematisk Afdeling 7:285–487.

[CIT0011] Stamatakis A 2014 RAxML version 8: a tool for phylogenetic analysis and post-analysis of large phylogenies. Bioinformatics. 30:1312–1313. 24451623 10.1093/bioinformatics/btu033 PMC3998144

[CIT0012] Zbawicka M, Wenne R, Burzyński A 2014 Mitogenomics of recombinant mitochondrial genomes of Baltic Sea *Mytilus* mussels. Mol Genet Genomics. 289:1275–1287. 25079914 10.1007/s00438-014-0888-3 PMC4236608

